# Determinants of Long-Term Care Services among the Elderly: A Population-Based Study in Taiwan

**DOI:** 10.1371/journal.pone.0089213

**Published:** 2014-02-19

**Authors:** Chen-Yi Wu, Hsiao-Yun Hu, Nicole Huang, Yi-Ting Fang, Yiing-Jeng Chou, Chung-Pin Li

**Affiliations:** 1 Institute of Public Health, National Yang-Ming University, Taipei, Taiwan; 2 Department of Dermatology, Taipei City Hospital, Heping Fuyou Branch, Taipei, Taiwan; 3 Department of Education and Research, Taipei City Hospital, Taipei, Taiwan; 4 Institute of Hospital and Health Care Administration, National Yang-Ming University, Taipei, Taiwan; 5 Institute of Health and Welfare Policy, National Yang-Ming University, Taipei, Taiwan; 6 Universal Eye Center, Taoyuan Branch, Taiwan; 7 Division of Gastroenterology, Department of Medicine, Taipei Veterans General Hospital, Taipei, Taiwan; 8 National Yang-Ming University School of Medicine, Taipei, Taiwan; “Mario Negri” Institute for Pharmacological Research, Italy

## Abstract

**Objectives:**

The aim of the study was to investigate determinants of long-term care use and to clarify the differing characteristics of home/community-based and institution-based services users.

**Design:**

Cross-sectional, population-based study.

**Setting:**

Utilizing data from the 2005 National Health Interview Survey conducted in Taiwan.

**Participants:**

A national sample of 2,608 people (1,312 men, 1,296 women) aged 65 and older.

**Measurements:**

The utilization of long-term care services (both home/community- and institution-based services) was measured. A χ^2^ analysis tested differences in baseline characteristics between home/community-based and institution-based long-term care users. The multiple-logistic model was adopted with a hierarchical approach adding the Andersen model’s predisposing, enabling, and need factors sequentially. Multiple logistic models further stratified data by gender and age.

**Results:**

Compared with users of home/community-based care, those using institution-based care had less education (p = 0.019), greater likelihood of being single (p = 0.001), fewer family members (p = 0.002), higher prevalence of stool incontinence (p = 0.011) and dementia (*P* = .025), and greater disability (p = 0.016). After adjustment, age (compared with 65–69 years; 75–79 years, odds ratio [OR] = 2.08, p = 0.044; age ≥80, OR = 3.30, p = 0.002), being single (OR = 2.16, p* = *0.006), urban living (OR = 1.68, p = 0.037), stroke (OR = 2.08, p = 0.015), dementia (OR = 2.32, p = 0.007), 1–3 items of activities of daily living (ADL) disability (OR = 5.56, p<0.001), and 4–6 items of ADL disability (OR = 21.57, p<0.001) were significantly associated with long-term care use.

**Conclusion:**

Age, single marital status, stroke, dementia, and ADL disability are predictive factors for long-term care use. The utilization was directly proportional to the level of disability.

## Introduction

The escalating growth of the elderly population in Taiwan and worldwide is of increasing concern. This population, defined as individuals aged 65 years or older, is projected to increase from 11% of the population in 2010 to 20% by 2025, with Taiwan becoming an aged nation according to World Health Organization (WHO) criteria [1]. The elderly, making up 6.9%–12.0% of the global population, have a higher incidence of chronic disease, and, as such, the aging population will present a higher prevalence of functional disability, subsequently leading to a greater utilization of long-term care services [2]. Such services are often required from onset of the condition for the remaining lifetime, making it of great important to better understand the factors associated with long-term care use to provide appropriate care and alleviate the societal burden caused by population aging.

In Taiwan, over 97% of health care is covered by the National Health Insurance (NHI); however, this does not include long-term care insurance. Although the NHI reimburses some home-care nursing services, recipients of such care must be severely disabled to qualify [3]. Nine per 1,000 elderly patients in Taiwan reportedly received home-care services in 2004, a rate much lower than the proportion of disabled older people in Taiwan [1]. For the severely disabled and for low-income families, there are some extended home services provided by the Departments of Health or Social Welfare of local governments.

Due to the heterogeneity of chronic diseases and disability, varying types of services are needed in long-term care. Long-term care can be roughly classified into two types: home/community-based and institution-based care. Elderly people’s disability status is reportedly the main factor driving the demand for long-term care services [4]. For example, cognitive impairments and reduction in activities of daily living (ADL) are key predictors of institutionalization [5], [6]. Further, home-visit nursing services utilization is lower when elderly people have no caregivers or are from low income households [7]. In Taiwan, old age, cognitive impairment, and functional disabilities are key predictors of the need for formal care, just as observed in Western countries [8]. However, most studies have focused on either home or institutional care [7], [9]–[14]; few have investigated simultaneous utilization of institutional and home/community care [15], [16]. Van Campen *et al.* used a multinomial model to analyze the use of long-term care packages in Netherlands [16]. But when close substitutes were noted among long-term care services, the independence of irrelevant alternatives assumption of the multinomial model was considered violated [15]. Meijer *et al.* investigated long-term care utilization using an ordered response model that assumed the hierarchical ordering of long-term care services [15]. However, with no long-term care insurance and limited publicly financed long-term care in Taiwan, choice of long-term care services depended on individuals’ preferences related to both socio-economic and need factors. Because these two factor types are not mutually exclusive, the multinomial and ordered response models were inappropriate analyses. Further descriptive data and investigations of different characteristics of care users may provide better insight into long-term care utilization distributions.

Some claims-based data do not include patient-reported factors, such as living environment, geriatric conditions, or functional disability, and therefore may overlook information important for assessing the true state of the elderly. For example, informal care may potentially substitute for formal home care and generally postpones long-term care use [17]. Co-residence status is associated with age, and was used as a proxy for informal care availability [18]. Among the elderly, geriatric conditions are similar in prevalence to chronic diseases [19]. Thus, information about individual situations is very important in investigations of long-term care use. Given improved data availability, we investigated the determinants of long-term care use and sought to clarify differences in the characteristics of home/community-based and institution-based service users.

## Materials and Methods

### Data Sources and Samples

The data for this population-based study were obtained from the National Health Interview Survey (NHIS) conducted in 2005 in Taiwan. NHIS participants were selected from the Taiwanese population using a multistage, stratified, systematic sampling design by geographic location and degree of urbanization. All individuals selected were interviewed by well-trained personnel using standardized questionnaires. A proxy participant was interviewed if the participant was unable to answer the questions. The NHIS data are available for public use and can be accessed at http://nhis.nhri.org.tw. A total of 24,726 persons completed the survey, of whom 2,727 individuals were aged 65 years or older; the overall response rate was 80.6%. Some participants were excluded due to missing data, and therefore we analyzed data from 2,608 (95.4%) elderly people. The data were weighted to achieve a nationally representative sample. Participants’ identification numbers were encrypted before the data were released for research purposes, ensuring that no participants could be identified. The survey complied with the Declaration of Helsinki, and received the approval of the institutional review board of the National Yang-Ming University (IRB No. 101003).

### Outcome Variable

Long-term care services consisted of publicly or privately financed care, and were classified into home/community-based and institution-based services. The former included formal domestic care, personal care, and home nursing care as well as temporary day care or community respite care, while the latter included admissions to residential or nursing homes. Residential homes provide assistance with domestic tasks, whereas nursing homes provide personal and nursing care. Because the survey question was “Did you ever use these long-term care services in the past year?” we could not differentiate whether the services were publicly or privately financed; further, as anticipated, home/community-based and institution-based services were not mutually exclusive.

### Independent Variables

Andersen’s Behavioral Model was used to investigate the associations between individual factors between long-term care services [20]. Variables were classified into predisposing, enabling, and need factors.

#### Predisposing factors

The predisposing factors we included were age, gender, education level, smoking (yes/no), current alcohol consumption (yes/no), and body mass index (BMI). Age was categorized as 65–69, 70–74, 75–79, and ≥80 years; education level as illiteracy, 1–6 years education, and ≥7 years education; and BMI (based on WHO criteria) as <18.5 kg/m^2^ (underweight), 18.5–24.9 kg/m^2^ (normal), and ≥25 kg/m^2^ (overweight).

#### Enabling factors

The enabling factors were marital status, co-residence with family members, household monthly income, and residence. Marital status was categorized as single or married/cohabiting; co-residence with family members as 0, 1–3, and ≥4 family members; household monthly income as <30,000, 30,000–69,999, and ≥70,000 NTD (1 USD = 30 NTD); and residence as either urban or non-urban.

#### Need factors

The need factors were self-reported geriatric conditions, chronic diseases, visiting a hospital emergency room in the past year, hospitalization in the past year, and disability status. Geriatric conditions included urine or stool incontinence. Chronic diseases were physician-diagnosed diseases including hypertension, diabetes, hyperlipidemia, stroke, heart disease, cancer, dementia, hearing problems, and vision problems. A history of visiting the emergency room or hospitalization in the past year was used as proxies of disease severity. ADL measured the difficulty in performance on six activities: eating, bathing, dressing, using the toilet, getting in or out of bed, and walking indoors [21]. The Instrumental Activities of Daily Living (IADL) measured the difficulty in performance on eight activities: cooking, buying groceries, operating a telephone, taking medications, household chores, laundering, cleaning the house, and managing personal finances [22]. According to the measured items in difficulty of performing each activity of ADL or IADL, disability status was then categorized as no disability, IADL disability only, 1–3 items of ADL disability, and 4–6 items of ADL disability.

### Statistical Analysis

Using a χ^2^ analysis, we examined differences in baseline characteristics between home/community-based and institution-based long-term care users. Andersen model was incorporated in the multiple logistic models, with hierarchical approach by added predisposing, enabling, and need factors sequentially. The outcome of the multiple logistic regression models was utilization of long-term care (home/community-based and institution-based). Multiple logistic models were further stratified by gender and age. All analyses were conducted using SAS 9.1 (SAS Institute Inc., Cary, NC) and STATA 10.0 (StataCorp LP, College Station, TX) and incorporated the weighted procedures used in the NHIS sampling design.

## Results

### Participant Characteristics

Participants’ characteristics (n = 2,608; age ≥65 years; Male = 50.3%, Female = 49.7%) are shown in Table 1. There were significant gender differences in education level (p<0.001), smoking (p<0.001), alcohol consumption (p<0.001), BMI (p = 0.003), marital status (p<0.001), existence of urine incontinence (p<0.001), hypertension (p = 0.017), diabetes (p = 0.004), hyperlipidemia (p* = *0.021), dementia (p = 0.008), and disability level (p<0.001).

**Table 1:** Characteristics of the study population.

Variables	Total population	Male	Female	p
	(N = 2,608)	(n = 1,312; 50.3%)	(n = 1,296; 49.7%)	
	%	%	%	
**Predisposing factors**				
Age (mean, 95% CI)	74 (73.7–74.3)	74.1 (73.7–74.5)	74 (73.6–74.4)	0.364
Age (years)				
65–69	32.2	15.4	16.8	0.073
70–74	27.3	13.8	13.5	
75–79	22.6	12.5	10.1	
≥80	18.1	8.6	9.4	
Education level (years)				
Illiteracy (0)	33.5	7.4	26.1	<0.001
1–6 years	43.8	25.9	17.9	
≥7 years	22.7	17	5.7	
Smoking	27.2	25.4	1.8	<0.001
Alcohol consumption	20.2	16.5	3.7	<0.001
BMI^a^ (2,603 persons)				
<18.5 (underweight)	6.4	2.8	3.6	0.003
18.5–25 (normal)	55.6	29.9	25.8	
≥25 (overweight)	38.1	17.6	20.4	
**Enabling factors**				
Marital status				
Single	35.9	10.9	25	<0.001
Married/cohabiting	64.1	39.4	24.7	
Family members				
No family members	9	4.6	4.4	0.185
1–3	49.1	25.6	23.5	
≥4	41.9	20.1	21.8	
Household monthly income (NTD)				
<30,000	50.9	25.9	25	0.563
30,000–69,999	29.2	14.8	14.4	
≥70,000	10.8	5.5	5.3	
Missing data	9.1	4.1	5	
Residence				
Urban	47	25.9	27.1	0.198
Non-urban	53	24.4	22.6	
**Need factors**				
Geriatric conditions				
Urine incontinence	23.8	7.8	16	<0.001
Stool incontinence	7.6	3.3	4.3	0.099
Chronic diseases				
Hypertension	42.4	20	22.4	0.017
Diabetes	17.1	7.4	9.7	0.004
Hyperlipidemia	21.9	9.9	12	0.021
Stroke	7.2	4	3.1	0.137
Heart disease	19.2	8.9	10.3	0.096
Cancer	2.6	1.3	1.3	0.885
Dementia	4.4	1.6	2.8	0.008
Hearing problems	18.4	9.5	8.9	0.526
Vision problems	5.4	3.1	2.3	0.117
Visited ER in the past year	20.3	9.9	10.4	0.543
Hospitalized in the past year	18.3	9.3	9	0.775
Disability				
No disability	58.6	34.8	23.8	<0.001
IADL disability only	27.6	10.4	17.2	
1–3 items of ADL disability	6	2.1	3.9	
4–6 items of ADL disability	7.8	3	4.8	

a Missing BMI data from 5 participants; BMI = body mass index; NTD = New Taiwan dollar; ER = emergency room; IADL = Instrumental Activities of Daily Living; ADL = Activities of Daily Living.

### Characteristics by Long-term Care Service Type

Table 2 presents participant characteristics grouped by non-long-term care users, and long-term care user (including both home/community- and institution-based care). During the year preceding assessment, 123 individuals (4.7%) used home/community-based services (home care, n = 22; paid caregiver, n = 98, [82 non-Taiwanese caregivers and 16 Taiwanese caregivers]; nursing care, n = 13; day care, n = 2; respite care, n = 3), and 68 persons (2.6%) used institution-based services. Compared with users of home/community-based care, those who used institution-based care had significantly lower education level (p = 0.019), being single (p = 0.001), fewer family members (p* = *0.002), and greater prevalence of stool incontinence (p = 0.011), dementia (p = 0.025), and disability (p = 0.016).

**Table 2:** Characteristics of the study population according to different long-term care use.

Variables	Non long-term care user		Long-term care user (n = 179, 6.9%)				P
			Home/community-based		Institution-based		
	(n = 2,429, 93.1%)		(n = 123, 4.7%)		(n = 68, 2.6%)		
	n	%	n	%	n	%	
**Predisposing factors**							
Age (mean, 95% CI)	73.6	73.3–73.8	79.1	77.7–80.6	78.3	76.4–80.2	0.252
Age							
65–69 years	821	33.8	15	12.1	5	7.7	0.264
70–74 years	678	27.9	22	18.1	14	20.4	
75–79 years	543	22.4	23	19	21	31.1	
≥80 years	386	15.9	62	50.8	28	40.8	
Gender							
Female	1,195	49.2	75	60.9	35	51.5	0.203
Male	1,234	50.8	48	39.1	33	48.6	
Education level							
Illiteracy	787	32.4	55	45	35	51.8	0.019
1–6 years	1,088	44.8	32	25.8	25	36.7	
≧7 years	554	22.8	36	29.3	8	11.5	
Smoking	671	27.6	24	19.2	16	23.3	0.514
Alcohol consumption	517	21.3	7	5.5	4	6.2	0.957
BMI*							
<18.5 (underweight)	137	5.6	14	11.4	14	21.5	0.192
18.5–25 (normal)	1,356	55.9	66	54	31	47	
≥25 (overweight)	932	38.4	43	34.7	21	31.5	
**Enabling factors**							
Marital status							
Single	822	33.8	71	57.5	55	80.2	0.001
Married/cohabiting	1,607	66.2	52	42.5	13	19.8	
Family members							
No family members	220	9.1	3	2.6	11	15.8	0.002
1–3	1,207	49.7	53	43.2	26	38.3	
≥4	1,002	41.3	67	54.2	31	45.9	
Household monthly income (NTD)*							
<30,000	1,251	51.5	51	41.8	32	47.4	0.882
30,000–69,999	705	29	39	31.8	20	29.9	
≥70,000	261	10.8	15	12	8	11.6	
Missing data	211	8.7	18	14.5	8	11.1	
Residence							
Urban	1127	46.4	69	55.7	40	58.8	0.671
Non-urban	1,302	53.6	55	44.4	28	41.3	
**Need factors**							
Geriatric conditions							
Urine incontinence	516	21.2	67	54.3	45	66.8	0.116
Stool incontinence	128	5.3	42	34.3	36	52.4	0.011
Chronic diseases							
Hypertension	1,009	41.6	67	54.8	35	51.7	0.691
Diabetes	393	16.2	34	27.6	21	30.5	0.636
Hyperlipidemia	531	21.9	31	25.4	10	15.3	0.091
Stroke	126	5.2	37	30.2	28	41.8	0.121
Heart diseases	468	19.3	25	20	9	13	0.22
Cancer	62	2.5	7	5.6	0	0	
Dementia	58	2.4	33	26.7	29	42.7	0.025
Hearing problems	418	17.2	42	34.1	19	27.9	0.378
Visions problems	131	5.4	7	5.5	7	9.8	0.243
Visiting ER in the past year	447	18.4	64	52.3	27	39.6	0.102
Hospitalized in the past year	397	16.3	56	45.6	32	47.6	0.839
Disability							
No disability	1,506	62	15	12.6	9	13.1	0.016
IADL disability only	698	28.7	18	14.7	4	6.1	
1–3 items of ADL disability	133	5.5	22	17.7	4	6.1	
4–6 items of ADL disability	93	3.8	68	55	51	74.7	?

*Missing 4 BMI data in non long-term care user; Missing 2 BMI data in institution user.

BMI: Body mass index; NTD: New Taiwan dollar; ER: emergency room; IADL: instrumental activities of daily living;

ADL: activities of daily living.

### Factors Related to Long-term Care Utilization

The statistics for long-term care service utilization are shown in Table 3. The adjusted Model 1, with predisposing factors entered, showed that older age (compared with age 65–69 years: age 70–74, odds ratio [OR] = 2.31, p = 0.01; age 75–79, OR = 3.62, p<0.001; age ≥80, OR = 8.10, p<0.001), and BMI <18.5 (OR = 2.44, p = 0.002, compared with normal BMI 18.5*–*25) were significantly associated with greater long-term care use. However, education level of 0–6 years (OR = 0.58, p = 0.023, compared with illiteracy), and alcohol consumption (OR = 0.32, p = 0.003) showed significant associations with reduced long-term care use.

**Table 3:** Multiple logistic regressions of long-term care use and related factors.

	Model 1		Model 2		Model 3	
Variables	OR	p	OR	p	OR	p
**Predisposing factors**						
Age (reference: 65–69 years)						
70–74	2.31	0.010	2.31	0.011	1.94	0.087
75–79	3.62	<0.001	3.07	<0.001	2.08	0.044
≥80	8.10	<0.001	5.75	<0.001	3.30	0.002
Gender (Female)						
Male	1.03	0.893	1.50	0.088	1.68	0.100
Education level (Illiteracy)						
1–6 years	0.58	0.023	0.58	0.028	0.65	0.165
≥7 years	0.78	0.382	0.75	0.338	1.25	0.590
Smoking (No)						
Yes	1.01	0.962	0.95	0.852	1.24	0.480
Alcohol consumption (No)						
Yes	0.32	0.003	0.32	0.002	0.47	0.085
BMI (18.5–25)						
<18.5	2.44	0.002	2.41	0.003	0.89	0.751
≥25	1.00	0.991	1.00	0.992	0.89	0.658
**Enabling factors**						
Marital status (married/cohabiting)						
Single			2.51	<0.001	2.16	0.006
Family members (no family members)						
1–3			1.59	0.231	1.00	0.992
≥4			1.99	0.078	1.49	0.320
Household monthly income (<30,000 NTD)						
30,000–69,999			1.11	0.636	0.81	0.414
≥70,000			1.10	0.772	0.69	0.306
Missing data			1.71	0.082	2.21	0.063
Residence (non-urban)						
Urban			1.55	0.024	1.68	0.037
**Need factors**						
Geriatric conditions						
Urine incontinence					1.67	0.061
Stool incontinence					1.36	0.294
Chronic diseases						
Hypertension					1.60	0.069
Diabetes					1.23	0.414
Hyperlipidemia					1.13	0.659
Stroke					2.08	0.015
Heart disease					0.50	0.025
Cancer					1.04	0.958
Dementia					2.32	0.007
Hearing problems					0.89	0.669
Vision problems					0.94	0.904
Visited ER in the past year					1.23	0.510
Hospitalized in the past year					1.56	0.195
Disability (no disability)						
IADL disability only					1.20	0.635
1–3 items of ADL disability					5.56	<0.001
4–6 items of ADL disability					21.57	<0.001

OR = odds ratio; BMI = body mass index; NTD = New Taiwan dollar; ER = emergency room;

IADL = Instrumental Activities of Daily Living; ADL = Activities of Daily Living.

Both predisposing and enabling factors were entered into the adjusted Model 2. Results for the predisposing factor were similar to the Model 1 findings. Among enabling factors, being single (OR = 2.51, p<0.001, compared with married/cohabiting), and living in urban regions (OR = 1.55, p = 0.024, compared with living in non-urban regions) were associated with significantly greater long-term care use.

In the adjusted Model 3, predisposing, enabling, and need factors were entered simultaneously. Age (compared with age 65–69 years: age 75–79, OR = 2.08, p = 0.044; age ≥80, OR = 3.30, p = 0.002), being single (OR = 2.16, p = 0.006, compared with married/cohabiting), living in urban regions (OR = 1.68, p = 0.037, compared with living in non-urban region), stroke (OR = 2.08, p* = *0.015), dementia (OR = 2.32, p* = *0.007), 1–3 items of ADL disability (OR = 5.56, p<0.001), and 4–6 items of ADL disability (OR = 21.57, p<0.001) were significantly associated with greater long-term care use. However, heart disease (OR = 0.50, p = 0.025) was associated with significantly reduced long-term care use.

Interactions terms (age × gender, age × marital status, gender × marital status) were added separately into Model 3; none were statistically associated with long-term care use (data not shown).

### Gender and Long-term Care Utilization

Table 4 shows results for utilization of long-term care when stratified by gender. Among men, older age (compared with age 65–69 years; age 75–79, OR = 3.93, p = 0.016; age ≥80, OR = 4.58, p = 0.022), being single (OR = 2.78, p = 0.033, compared with married/cohabiting), living in urban regions (OR = 2.41, p = 0.032, compared with living in non-urban regions), stool incontinence (OR = 2.71, p = 0.027), dementia (OR = 4.73, p = 0.009), and 4–6 items of ADL disability (OR = 15.14, p<0.001) were all significantly associated with greater long-term care use; education level of 0–6 years (OR = 0.30, p = 0.015, compared with illiteracy) and alcohol consumption (OR = 0.24, p = 0.023) showed significant associations with reduced long-term care use. Among women, older age (compared with age 65–69 years; age ≥80, OR = 2.98, p = 0.036), education level ≥7 years (OR = 3.62, p = 0.03, compared with illiteracy), being single (OR = 2.24, p = 0.023, compared with married/cohabiting), hypertension (OR = 2.46, p = 0.006), stroke (OR = 2.78, p = 0.013), dementia (OR = 2.70, *P* = .016), 1–3 items of ADL disability (OR = 16.51, p<0.001), and 4–6 items of ADL disability (OR = 42.95, p<0.001) were all significantly associated with greater long-term care use; heart disease (OR = 0.39, p = 0.039) showed a significant association with reduced long-term care use.

**Table 4:** Multiple logistic regressions of long-term care use stratified by gender.

	Male		Female	
Variables	OR	p	OR	p
**Predisposing factors**				
Age (reference: 65–69 years)				
70–74 years	2.30	0.221	1.94	0.196
75–79 years	3.93	0.016	1.28	0.599
≥80 years	4.58	0.022	2.98	0.036
Education level (Illiteracy)				
1–6 years	0.30	0.015	1.05	0.908
≥7 years	0.45	0.160	3.62	0.030
Smoking (No)				
Yes	1.93	0.099	0.15	0.054
Alcohol consumption (No)				
Yes	0.24	0.023	1.03	0.958
BMI (18.5–25)				
<18.5	0.77	0.668	0.87	0.759
≥25	0.69	0.457	1.06	0.876
**Enabling factors**				
Marital status (married/cohabiting)				
Single	2.78	0.033	2.24	0.023
Family members (no family members)				
1–3	1.86	0.380	0.61	0.379
≥4	1.95	0.345	1.20	0.723
Household monthly income(<30,000 NTD)				
30,000–69,999	0.50	0.078	0.99	0.979
≥70,000	0.35	0.206	1.02	0.968
Missing data	1.36	0.676	2.77	0.066
Residence (non-urban)				
Urban	2.41	0.032	1.43	0.276
**Need factors**				
Geriatric conditions				
Urine incontinence	1.97	0.134	1.24	0.554
Stool incontinence	2.71	0.027	0.87	0.728
Chronic diseases				
Hypertension	1.00	0.991	2.46	0.006
Diabetes	1.89	0.217	1.06	0.835
Hyperlipidemia	1.92	0.152	0.67	0.313
Stroke	1.80	0.212	2.78	0.013
Heart disease	0.73	0.515	0.39	0.039
Cancer	2.70	0.225	0.75	0.778
Dementia	4.73	0.009	2.70	0.016
Hearing problems	1.24	0.612	0.80	0.540
Vision problems	1.57	0.615	1.07	0.937
Visited ER in the past year	1.99	0.198	1.18	0.69
Hospitalized in the past year	1.27	0.678	1.95	0.107
Disability (no disability)				
IADL disability only	0.96	0.935	1.05	0.937
1–3 items of ADL disability	1.36	0.677	16.51	<0.001
4–6 items of ADL disability	15.14	<0.001	42.95	<0.001

OR = odds ratio; BMI = body mass index; NTD = New Taiwan dollar; ER = emergency room; IADL = Instrumental Activities of Daily Living; ADL = Activities of Daily Living.

### Age and Long-term Care Utilization

Table 5 shows the analysis stratified by two age groups: young-old elderly (65–74 years) and old-old elderly (≥75 years). Among young-old elderly, male gender (OR = 2.89, p = 0.049, compared with female) and 4–6 items of ADL disability (OR = 28.17, p<0.001) were significantly associated with greater long-term care use; alcohol consumption (OR = 0.12, p = 0.011) and low BMI (OR = 0.15, p = 0.017) showed significant associations with reduced long-term care use. Among old-old elderly, being single (OR = 2.68, p = 0.012, compared with married/cohabiting), stroke (OR = 3.52, p = 0.002), dementia (OR = 3.50, p = 0.001), 1–3 items of ADL disability (OR = 7.18, p = 0.001), and 4–6 items of ADL disability (OR = 29.15, p<0.001) were significantly associated with greater long-term care use; heart disease (OR = 0.37, p = 0.015) showed a significant association with reduced long-term care use.

**Table 5:** Multiple logistic regressions of long-term care use stratified by age.

	Young-old (aged 65–74 years)		Old-old (aged ≥75 years)	
Variables	OR	p	OR	p
**Predisposing factors**				
Gender (Female)				
Male	2.89	0.049	1.59	0.238
Education level (Illiteracy)				
1–6 years	0.57	0.366	0.55	0.146
≥7 years	0.31	0.143	2.20	0.149
Smoking (No)				
Yes	0.97	0.950	1.76	0.131
Alcohol consumption (No)				
Yes	0.12	0.011	0.46	0.221
BMI(18.5–25)				
<18.5	0.15	0.017	1.29	0.583
≥25	0.71	0.491	1.07	0.854
**Enabling factors**				
Marital status (married/cohabiting)				
Single	2.29	0.099	2.68	0.012
Family members (No family members)				
1–3	3.10	0.212	0.57	0.259
≥4	3.79	0.144	1.17	0.753
Household monthly income(<30,000 NTD)				
30,000–69,999	1.51	0.372	0.67	0.221
≥70,000	1.08	0.898	0.85	0.713
Missing data	2.10	0.304	2.15	0.139
Residence (non-urban)				
Urban	1.88	0.186	1.61	0.119
**Need factors**				
Geriatric conditions				
Urine incontinence	2.09	0.250	1.35	0.340
Stool incontinence	1.60	0.350	0.98	0.967
Chronic diseases				
Hypertension	1.70	0.283	1.38	0.346
Diabetes	2.03	0.135	0.90	0.741
Hyperlipidemia	0.71	0.53	1.43	0.335
Stroke	0.71	0.582	3.52	0.002
Heart disease	0.75	0.587	0.37	0.015
Cancer	1.42	0.788	0.74	0.736
Dementia	1.51	0.539	3.50	0.001
Hearing problems	1.17	0.749	0.89	0.732
Vision problems	3.88	0.075	0.44	0.255
Visited ER in the past year	1.79	0.303	0.80	0.589
Hospitalized in the past year	2.70	0.128	1.44	0.362
Disability (no disability)				
IADL disability only	0.90	0.858	1.39	0.530
1–3 items of ADL disability	5.35	0.081	7.18	0.001
4–6 items of ADL disability	28.17	<0.001	29.15	<0.001

OR = odds ratio; BMI = body mass index; NTD = New Taiwan dollar; ER = emergency room;

IADL = Instrumental Activities of Daily Living; ADL = Activities of Daily Living.

## Discussion

We used a nationwide representative sample to investigate the determinants of long-term care utilization among the elderly of Taiwan. Older age, stroke, dementia, ADL disability, and single marital status best predicted long-term care use. Utilization was directly proportional to the level of ADL disability, independent of geriatric conditions or chronic diseases. Furthermore, compared with home/community-based care, users of institution-based care were less educated, had fewer family members, were more disabled, and were more likely to be single, to have dementia, and to experience stool incontinence.

In the present study sample, this ratio of home/community-based to institution-based services was 2∶1 (4.7% vs. 2.6%), which is compatible with findings from other studies; however, the usage rates were still much lower than those of countries with insurance-covered long-term care services, such as the Netherlands (15.7%; home care, 12.7%; institutional care, 4.5%) or Japan (13.1%) [4], [9]. Utilization of long-term care services may be significantly underestimated due to under-reporting and lack of universal long-term care service coverage. Considering that 13.8% of the elderly in our sample had at least one item of ADL disability and 7.8% had 4–6 items, the long-term care usage rate of 6.9% in Taiwan suggests that informal care is a partial substitute for formal care. Without insurance coverage, accessibility to long-term care may become a concern, and put considerable burden on informal (usually family) caregivers.

We observed no significant age difference by gender; however, the women in our study were significantly more disabled, had more geriatric conditions, and greater prevalence of hypertension, diabetes, hyperlipidemia, and dementia than did the men. This is consistent with previous research [11], [23]. Women make up a disproportionate number of the disabled elderly because they tend to live longer than men [23]. Furthermore, disabled women may be particularly vulnerable to unmet needs because many live alone and have limited resources [11]. They are more likely to be disabled through chronic disease than are men [24]. An interesting finding was that women with 1–3 items of ADL disability had dramatically increased odds of using long-term care; this may reflect the fact that women are more likely to be single and receive limited informal care from family members. Male gender was associated with a greater tendency to use long-term care, especially in the young-old group. Previous research has suggested male gender as a predictor for institutionalization, but the findings were inconsistent [6]. In short, it is important to take into account the disparity between genders in relation to health and social conditions when providing long-term care.

In Models 1 and 2 of our study, underweight was significantly associated with long-term care use. Underweight (BMI <18.5) is an international indicator of malnutrition and poor physical function. [25] Honda *et al.* reported that underweight may be a good predictor of long-term care in the young-old elderly [26]. However, in our study, after adding need factors, underweight was not associated with long-term care use. In fact, when geriatric conditions, chronic diseases, and disability were separately entered into Model 3, underweight was no longer a significant factor (data not shown). Furthermore, in the young-old group, underweight showed an association with reduced long-term care use. Therefore, when health status is controlled, underweight may play a less important role in long-term care usage than was previously thought.

After adjusting our model for disability, geriatric conditions and chronic disease (except stroke and dementia) were not significant contributors of long-term care use. However, chronic diseases did affect long-term care use by causing functional impairment. We defined disease severity as whether participants had visited an emergency room or been hospitalized; according to these measures, disease severity was not associated with long-term care use. After adjustment, dementia and stroke remained independently associated with long-term care use; 42.7% of institution users had dementia, whereas a significantly lower proportion (26.7%) of home/community based users had dementia. This is consistent with the understanding that dementia is a well-known contributor to long-term institutional usage [5], [6].

Functional disability among the elderly is generally assessed by impairments in ADL, as a key indicator of personal independence and criteria for provision of long-term care services [24]. The IADL has been used as a complementary index for measuring less severe levels of disability, including tasks that require higher level of personal autonomy [22]. The disability level in our sample was comparable with that found in previous studies [2], [27]. Disability indicated by the IADL was not associated with long-term care use, and we found a dose-response relationship between ADL disability and long-term care use.

In our study, when compared with home/community-based care users, institution-based care users were more likely to be single and to have fewer family members. Furthermore, single marital status was significantly associated with long-term care use, while co-residence with family members was not. Other researchers have found that informal care availability was approximated by marital status and co-residence with family members, and was noted to be a closer substitute for less skilled long-term care services [17]. Marital status is reportedly a significant predictor of skilled-nursing service utilization and ADL support [14], [28]. Availability of informal care may lessen the demand for long-term care [29]. Those elderly individuals living with spouses are less likely to use paid help than are those living with adult children [30]. These findings, together with our study, suggest that the spouse plays the dominant role in informal long-term care.

We note that our study had several limitations. First, the survey data were based on self-report; thus the severity of geriatric conditions, chronic diseases, and disability could not be directly measured. Second, the current study was cross-sectional but health status and disability are likely to be dynamic processes that change with time. A particular disadvantage was that measurement at a single time point precluded discussion of causality or the temporal relationship of service use. Third, our study design did not permit the collection of data regarding the causes of long-term care use. As long-term care services are not fully organized into a coherent system, and since most long-term care services are funded privately and out-of-pocket, the choice of whether to use services and what services to use is dependent on complex processes. Such data could only be collected by carrying out prospective cohort studies with planned data collection. Fourth, our study could not measure the level of informal care, that is, care provided by a spouse or relatives in the absence long-term care usage.

In conclusion, ADL disability was the most important factor in long-term care usage, with age, single marital status, stroke, and dementia also playing significant roles. Integrating these findings with those of others could help improve understanding of long-term care utilization among the elderly, which could help policymakers construct a better long-term care system for supporting the elderly and prepare them to be members of an aging society.
